# Opening-Wedge High Tibial Osteotomy with a Cancellous Strut Bone Allograft Is Inadequate for Achieving Satisfactory and Lasting Correction in Neglected Infantile Tibia Vara: Results from a Cohort of 29 Patients

**DOI:** 10.3390/jcm13144261

**Published:** 2024-07-22

**Authors:** Alessandro Depaoli, Marco Ramella, Grazia Chiara Menozzi, Giovanni Luigi Di Gennaro, Gino Rocca, Giovanni Trisolino

**Affiliations:** 1 Rizzoli Sicilia Department, IRCCS Istituto Ortopedico Rizzoli, 90011 Bagheria, Italy; alessandro.depaoli@ior.it; 2 Unit of Pediatric Orthopedics and Traumatology, IRCCS Istituto Ortopedico Rizzoli, 40136 Bologna, Italy

**Keywords:** infantile tibia vara, Blount’s disease, varus knee, tibia, child, opening wedge, high tibial osteotomy, osteotomy, allograft

## Abstract

**Background**: Infantile tibia vara (ITV) is a rare proximal tibia deformity in infancy, leading to progressive knee varus. High tibial osteotomy is commonly practiced but has high recurrence rates. This study analyzed factors affecting treatment failure and recurrence in children undergoing opening-wedge high tibial osteotomy (OWHTO) for ITV. **Methods**: We retrospectively studied children with ITV who had OWHTO with a press-fit cancellous bone allograft between 2000 and 2020, with ≥2-year follow-up. Outcomes included recurrence (knee varus with tibiofemoral angle > 10°), complications, and reintervention. **Results**: We analyzed 39 knees in 29 patients (mean age: 4.8 ± 1.9 years; median follow-up: 7.4 years). Recurrence occurred in 22 cases (56%). Age at surgery significantly influenced recurrence, with rates of 16% before age 5 versus 95% later (hazard ratio: 12.0, *p* = 0.001). Langenskiöld stage also affected recurrence (β-coefficient: 2.7, 95% C.I. 1.0–4.5, *p* = 0.002; pseudo-R-squared: 0.50, *p* = 0.001), with recurrence in all stage IV or higher cases. **Conclusions**: Early diagnosis and treatment before age 5, ideally with Langenskiöld stage III or lower, are crucial for stable correction with OWHTO alone. Late, high-grade ITV may require combined, acute or gradual, and/or staged correction. Further evidence is needed for optimal management.

## 1. Introduction

Infantile tibia vara (ITV), or Blount’s disease of infancy, presents as a rare, multiplanar deformity of the proximal tibia that develops in toddlers and preschoolers, characterized by a progressive accentuation of knee varus [1]. While factors like Afro-Caribbean ethnicity, obesity, and early walking onset have been linked to ITV pathogenesis, its exact cause remains unknown [2].

ITV must be distinguished from late-onset tibia vara (LOTV), characterized by normal knee development in childhood followed by tibial plateau growth arrest around ages 10–12 [2]. In contrast, ITV typically affects children in early years, often bilaterally, rapidly progressing to more severe disease manifestations, including depression of the medial tibial plateau, knee instability, and premature fusion of the medial proximal tibial physis, resulting in additional deformities such as proximal tibial procurvatum, internal tibial torsion, and limb length discrepancy, along with compensatory deformities in the distal femur and distal tibia [3]. If non-operative interventions fail, these deformities persist and worsen with skeletal maturity, potentially resulting in persistent pain, gait abnormalities, premature knee arthritis, and worsened limb length discrepancy [4]. Timely surgical intervention is crucial to mitigate these complications and improve prognosis [2].

Several articles have explored the optimal surgical options and timing for intervention in ITV treatment [5,6,7]. Currently, the most commonly performed surgical treatment for ITV is high tibial osteotomy, with various techniques described in the literature. Moreover, timely treatment, typically before the age of 4.5 years, is thought to enhance prognosis and reduce recurrence rates, regardless of the specific surgical technique employed [8].

The aim of this study was to investigate a cohort of children undergoing opening-wedge high tibial osteotomy (OWHTO) with a press-fit bone allograft for treating ITV. We aimed to analyze the recurrence rate, the time from surgery to recurrence onset, and potential predictive factors for failure and recurrence post-intervention.

## 2. Materials and Methods

### 2.1. Study Design and Inclusion and Exclusion Criteria

A retrospective analysis of skeletally immature patients affected by ITV who underwent the first surgical procedure between 2000 and 2020 was conducted. Inclusion criteria were: (1) age at surgery of less than 18 years, (2) diagnosis of ITV, (3) treated with an opening-wedge OWHTO performed in our institution between 1 January 2000 and 31 December 2020. The following exclusion criteria were applied: (1) patients with LOTV, (2) patients with clinical and/or radiographic evidence of genu varum due to any etiology different from ITV, (3) knees that underwent one or more surgical procedures for ITV in the period prior to that investigated by the study, (4) patients undergoing surgical procedures other than OWHTO with a cancellous strut bone allograft, (5) patients with less than 2 years of follow-up, and (6) cases lacking sufficient clinical or radiographic material for analysis.

### 2.2. Baseline Variables

Clinical baseline variables included: sex, side, body mass index (BMI), age at diagnosis, family history of ITV, comorbidities, clinical varus, lower limb length discrepancy (LLD), thigh–foot angle (TFA), and presence of symptoms. Patients were defined as obese if BMI was equal or greater than the 95th percentile adjusted by age and sex.

Preoperative radiographic images were retrieved and the following parameters were measured: stage according to Langenskiöld classification, mechanical axis distance in millimeters (MAD), the anatomic tibiofemoral angle (aTFA), the Drennan–Levine proximal tibial metaphyseal–diaphyseal angle (TMDA), the angle of depression of the medial tibial plateau (MPDA), the angle between the planes of the femoral condyles and the tibial shaft axis (femoral condyle to tibial shaft angle or FC–TS angle), and the conventionally applied angles to describe the alignment of the knee and distal tibia (mechanical lateral distal femoral angle or mLDFA, medial proximal tibial angle or MPTA, and lateral distal tibial angle or LDTA) [9,10,11,12]. Variables related to the surgical procedures included: age at surgery and the different angles of coronal angular correction, calculated as the difference between the FC–TS angle, the Drennan–Levine angle (or TDMA), the MPDA, and the MPTA measured on the immediate post-operative radiographic images and the preoperative values.

### 2.3. Outcome Variables

Recurrence and complications were considered outcome variables. Recurrence was defined as any radiographic and/or clinical evidence of genu varum (aTFA > 10°) that required any further surgical correction. Time free from recurrence was quantified as years between the surgical treatment and the first reported evidence of recurrence. Complications were evaluated according to the Clavien–Dindo–Sink classification modified by Dodwell et al. for pediatric orthopedic surgery [13]. Complications of grades I and II were categorized as minor, while complications of grades III up to V were defined as major. Further surgical procedures were also evaluated in patients who reached age 18 or older at the time of the analysis.

All patients were asked by telephone to fill an Italian version of the International Knee Documentation Committee (IKDC) Subjective Knee Form [14]. For patients younger than 16 years, the pediatric version (Pedi-IKDC) was given to the caregiver to complete together with the child [15].

### 2.4. Surgical Technique and Post-Operative Management

All procedures were performed with the patient in supine position with a pneumatic tourniquet at the root of the lower limb to be operated on. The contralateral limb was sometimes included in the sterile field for comparison, according to the preference of the surgeon.

**Figure 1:**
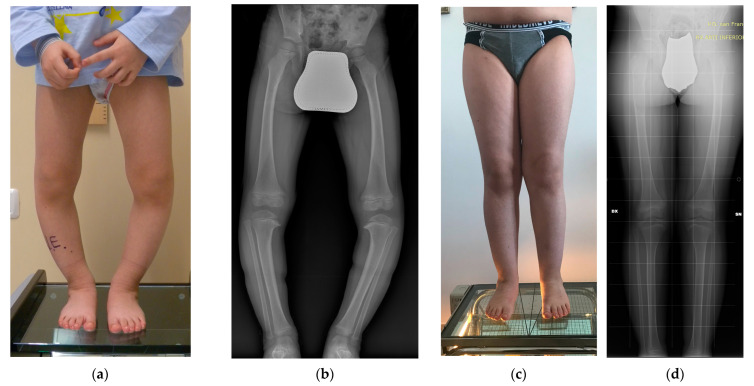

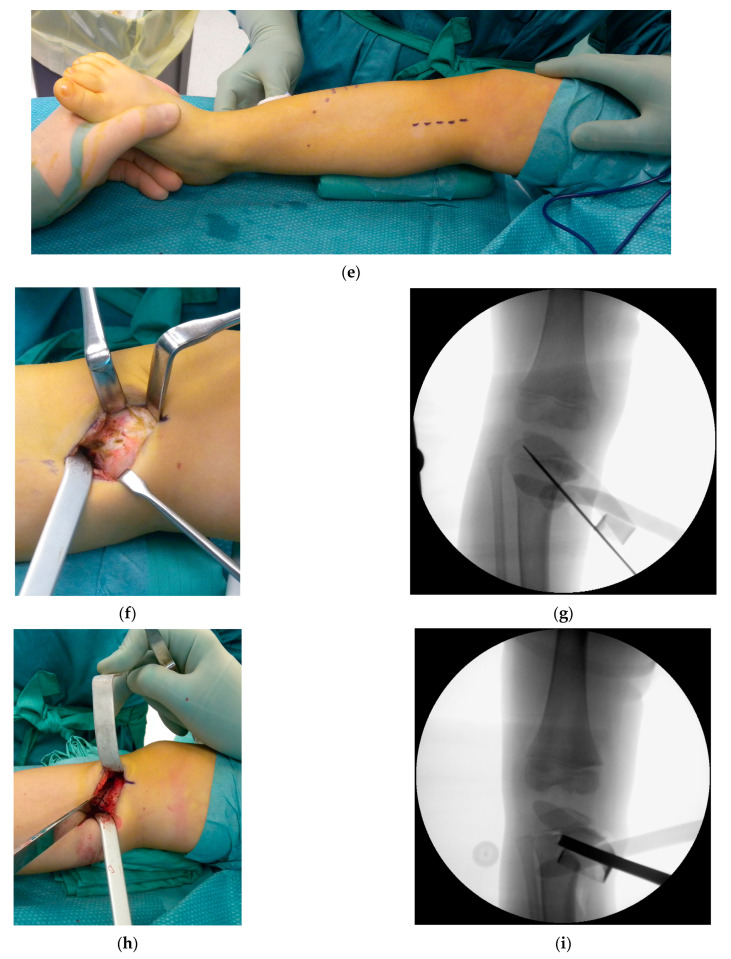

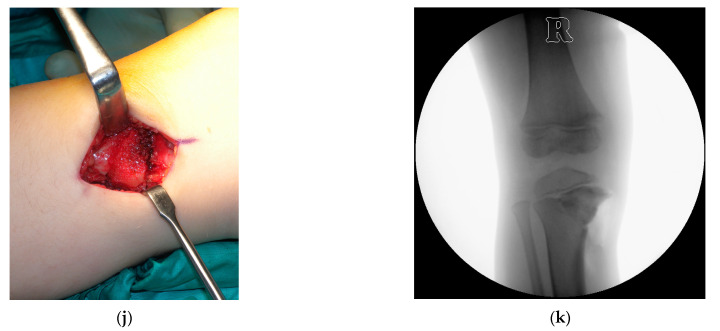
Case of a boy affected by bilateral ITV with intraoperative images of the surgical procedure on the right side: (**a**) clinical and (**b**) radiographic preoperative images at 3 years and 11 months of age; (**c**) clinical and (**d**) radiographic appearance at 10 years and 2 months of age (6 years and 3 months of follow-up) showed an optimal alignment of lower limbs; (**e**) draping of the right leg with the incision site marked on the skin; (**f**) medial side of the proximal tibia was subperiosteally exposed; (**g**) a 1.6 mm Kirschner wire was implanted aiming to the lateral border of the tibial plate and its position was checked under fluoroscopy; (**h**) an osteotomy was made with a chisel, avoiding a break through the physis, and (**i**) its position was checked with fluoroscopy; (**j**) final position of the bone graft and (**k**) its radiographic appearance.

A fibular osteotomy was made in all cases by removing a 5 mm cylinder of bone through an incision of approximately 2–3 cm between the distal third and fourth fibula. Subsequently, a linear incision of 4–5 cm on the medial side of the tibia was made to subperiosteally expose the medial portion of the tibia. With the help of fluoroscopy, a chisel opening-wedge osteotomy was performed, aiming the tip of the chisel at the lateral margin of the tibial plateau. Desired valgus angular correction was achieved on the coronal plane and maintained by fitting a wedge of cancellous strut bone allograft, produced at the domestic Bone Tissue Bank (Figure 1). Each plane of tissue was then sutured, leaving the fascia open to avoid compartment syndrome. In some cases, drainage was maintained in the deep tissues for the next 24 h.

All patients were immobilized in a long leg cast with the knee at about 30–40° of flexion for 6 weeks. After cast removal, swimming, resumption of active mobilization and progressive loading to full over the next month were allowed. No specific physiotherapy protocols were applied.

### 2.5. Data Analysis

Data were collected using Excel (Microsoft Corporation, Redmond, WA, USA) and then transferred to STATA (StataCorp. 2022. Stata Statistical Software: Release 17. College Station, TX, USA: StataCorp LLC.) to perform statistical analysis.

Continuous variables will be summarized in terms of mean and standard deviation (SD), if normally distributed according to Shapiro–Wilk test. Otherwise they will be summarized in terms of median and interquartile range (IQR, reported indicating the values of the first quartile and of the third quartile). Categorical or ordinal data will be summarized in terms of frequency and percentages. Spearman’s test for nonparametric variables was performed, and possible correlations that emerged were evaluated with subsequent uni- or multivariate linear regressions for values of *p* < 0.1. A comparison of discrete variables was assessed by constructing contingency tables and performing a chi-squared test or a Fisher’s exact test for small sample size groups. Visual analysis of data distribution was performed, and the STATA threshold regression model was applied to calculate the value of thresholds. A *p* < 0.05 was considered statistically significant.

## 3. Results

### 3.1. Demographic and Preoperative Clinical/Radiographic Variables

**Figure 2:**
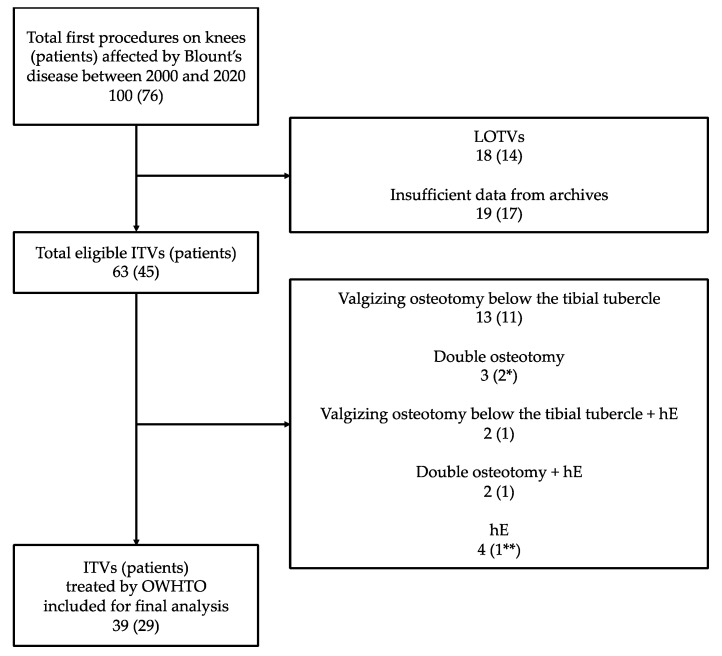
Flowchart representing patients’ inclusion process. ITV = infantile tibia vara; LOTV = late-onset tibia vara; hE = proximal tibia lateral hemiepiphysiodesis; OWHTO = opening-wedge high tibial osteotomy. (*) = one patient was not excluded, since the contralateral knee fit for inclusion; (**) = two patients were not excluded, since the contralateral knee of both fit for inclusion.

Between January 2000 and December 2020, 100 knees in 76 patients underwent the first surgical correction for Blount’s disease. From this initial pool, we excluded 14 patients (18 knees) with LOTV and 17 patients (19 knees) that had insufficient clinical and radiographic data. Among the remaining 45 patients (63 knees), 24 knees in 16 patients were excluded since they were treated with different techniques, such as valgizing osteotomy below the tibial tubercle, isolated hemiepiphysiodesis, or double osteotomy with or without hemiepiphysiodesis (see details in Figure 2). For the final analysis, we included 39 knees in 29 patients treated by OWHTO stabilized with a press-fit cancellous bone graft wedge.

Twenty patients (69%) were females. The condition was bilateral in 13 patients (45%). Nine of them were treated with an OWHTO in both knees, while four patients underwent different types of corrections on the contralateral side: a double osteotomy in one case and a lateral hemiepiphysiodesis of the proximal tibia in three cases. Those four knees were excluded from the final analysis (see Figure 2).

Overall, twenty-two patients (76%) were obese. Among them, six patients had also the following associated conditions:

- neurofibromatosis type 1 (one patient with bilateral involvement);
- microcytemia (one patient with bilateral involvement);
- post-axial polydactyly of both hands and both feet (one case);
- precocious puberty at 6 years of age (one case);
- GH-deficiency and hypocorticosurrenalism (one case);
- asthma (one case).

Among non-obese patients, only one case with bilateral involvement and knees treated with different techniques was associated with developmental dysplasia of the hip, which was treated with abduction splint during infancy. No association was found between obesity and bilateral involvement (*p* = 0.10).

The mean age at first evaluation of patients in our institute was 4.8 ± 1.9 years (range 2.1–9.4 years). Surgical correction was performed at a median age of 5.2 years old (IQR 4.1–7.4 years, range 3.2–9.9 years), and the median follow-up was 7.4 years (IQR 4.1–10.6 years, range 2.6–23.1 years). On average, surgery was performed 0.9 ± 0.6 years after the first evaluation in our institute (range 0.1–2.0 years).

Overall statistics of preoperative radiographic variables are reported in Table 1.

### 3.2. Surgical Outcome Variables

Recurrence was observed in 22 cases (56%) without difference in the average follow-up (*p* = 0.643). Median time to clinical and/or radiographic evidence of recurrence was 2.3 years (IQR 1.6–2.5 years).

A higher prevalence of recurrence was observed in females (see Table 1). However, females showed a higher mean age at surgery (6.1 ± 2.0 years compared with 4.7 ± 1.1 in males). Obesity and unilateral or bilateral involvement did not show any influence on prevalence of recurrence (*p* > 0.50).

**Figure 3:**
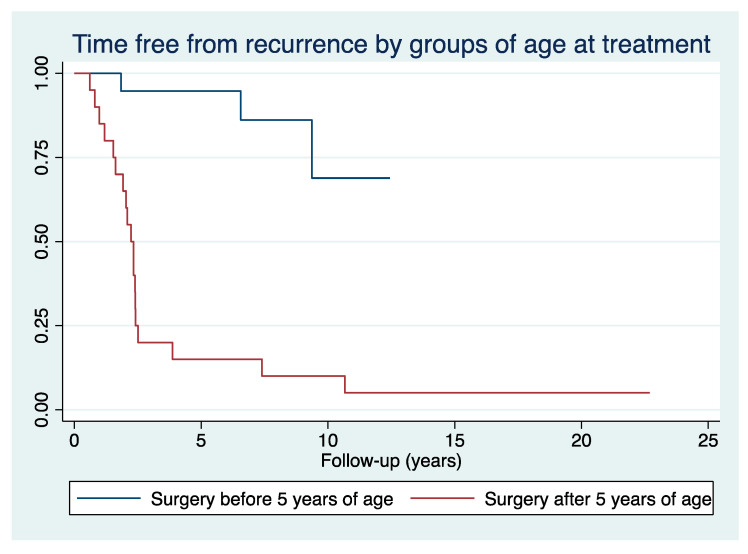
Kaplan–Meier survival function charts with patients divided into age groups at surgery (less than 5 years compared with 5 years or more).

Age at surgery showed a strong impact on recurrence rate (β-coefficient 2.0, 95% C.I. 0.6–3.4 with *p* = 0.005, pseudo-R^2^ 0.52 with *p* = 0.001). Children treated before 5 years of age had recurrence in 16% of cases, while children treated after 5 years of age had recurrence in 95% of cases (hazard-ratio 12.0, *p* = 0.001, see Figure 3). Moreover, 75% of cases treated after 5 years of age showed varus recurrence before 2.5 years of follow-up. It is noteworthy that the 10 knees treated on both sides with this technique had the same outcome in all cases but one. This patient was treated at 4.4 years on the right side (which was the most severely involved) and at 4.6 years on the contralateral side. Recurrence was documented on the right knee at 11.0 years of age, while no signs of recurrence were identified on the left side at the last available evaluation at 14.6 years of age (10.0 years of follow-up). One case with bilateral involvement was treated at 3.2 years of age on the right side and at 5.2 years on the left side and showed recurrence on both sides. All other 8 patients with bilateral involvement were treated in the same group of age at treatment and showed no recurrence in all cases before 5 years of age and recurrence in all cases after 5 years.

Among preoperative radiographic measures, Langenskiöld stage showed a significant influence on recurrence rate (β-coefficient 2.7, 95% C.I. 1.0–4.5 with *p* = 0.002, pseudo-R^2^ 0.50 with *p* = 0.001), with recurrence in all patients with a stage over III. MPTA, TMDA and FC–TS angle showed a weak influence on recurrence rate (pseudo-R^2^ 0.21 with *p* = 0.004, pseudo-R^2^ 0.18 with *p* = 0.004, R^2^ 0.15 with *p* = 0.012). No influence on recurrence was observed with MAD, aTFA, MPDA, mLDFA, or LDTA.

The extent of angular correction was comparable among patients with and without recurrence (*p* > 0.163). However, cases with recurrence had significantly more abnormal values of preoperative and postoperative TMDA, FC–TS angle, and MPTA (*p* < 0.021). The value of these angles on the postoperative radiographs showed a weak influence on the recurrence rate (pseudo-R^2^ 0.27, *p* = 0.008).

With the numbers available, age at treatment showed the strongest impact on recurrence, with negligible impact of preoperative angles (*p* > 0.11), postoperative angles (*p* > 0.48), and their difference (*p* > 0.59).

### 3.3. Complications

Overall, six minor complications (15%) and one major complication (3%) were observed. Minor complications were all mCDS grade 1 and included: two cases of slipping of the cast, which required it to be changed, one case of weakening of the cast, which required local repair, one superficial skin lesion of the heel after cast removal, which healed spontaneously, one case of skin rash in the postoperative period, which delayed by 1 month the surgical correction on the contralateral side, and one case of asymptomatic nonunion of the fibular osteotomy, which did not require treatment. A female with bilateral ITV was treated at 4.5 years of age on both sides and had a major complication on the knee with the less severe deformity, since it was hypercorrected into valgus (mCDS grade 3). The persistent valgus deformity required osteotomy at 11.2 years of age, while the contralateral side, initially with a more severe varus, showed recurrence of the varus deformity, which required revision at 12.6 years of age.

Of the 29 patients, 17 were reachable by telephone (59%); however, only 7 (24%, for a total of 9 knees) returned the completed IKDC or Pedi-IKDC questionnaire. Mean IKDC score was 77.3 ± 12.7 points.

All demographic and radiographic variables showed no influence on complication rate.

### 3.4. Surgical Revisions until Skeletal Maturity

At the time of the analysis, 26 cases (66%) had fully reached skeletal maturity. Among them, 7 knees (27%) maintained good alignment, while 18 cases of recurrence (69%) and 1 case of hypercorrection (4%) required one or more surgical revisions. Overall, each patient required a mean of 1.9 ± 0.7 surgical procedures to treat this condition. All the seven cases that required no revision were initially treated before 4 years of age.

A first revision surgery for recurrence was performed at a median age of 11.5 years (IQR 10.8–14.1 years). The same surgical procedure of osteotomy and a press-fit graft was performed in 4 cases (combined with medial femoral hemiepiphysiodesis in two knees), one case was treated with a hemiepiphysiodesis of the proximal lateral tibia with a tension-band plate, while the remaining 13 cases required high tibial osteotomy and plate fixation.

Among them, a second revision surgery was required in 6 cases (33%) at a median age of 12.5 years (IQR 11.9–13.8 years). All these cases were initially treated between 5 and 8 years of age and were all revised for the first time before 11.5 years of age. The surgical techniques chosen were: proximal tibia osteotomy with a press-fit graft and hemiepiphysiodesis of the lateral proximal tibia in one case, hemiepiphysiodesis in one case, and high tibial osteotomy and plate fixation in the other four cases. None of the patients required a third surgical revision.

The single case of hypercorrection was treated with a varus osteotomy below the tibial tuberosity and Blount’s staples fixation and it required no further surgical procedures.

## 4. Discussion

Our study presents medium to long-term outcomes of acute correction through OWHTO using a press-fit allograft in a large cohort of children with ITV, revealing a notable recurrence rate within just 2.5 years of follow-up. Despite the large number of variables considered, the present study confirmed that age at treatment appears to be the most important, if not the only, factor in determining the risk of recurrence in the surgical treatment of ITV. In our cohort, the recurrence rate was 16% before five years of age and 95% thereafter, highlighting the crucial role of immediate surgical intervention to minimize recurrence and reoperation risk. Over two-thirds of treated cases required additional surgical correction during childhood, with one-third needing more than two surgeries for achieving the desired correction. This is a crucial aspect, and parents should be informed early about the non-negligible possibility of multiple interventions during growth to achieve the final correction of the knee axis. In 1987, Ferriter and Shapiro reported 31% of recurrence in patients treated before four and a half years of age, compared with 76% after this threshold [16]. A higher rate of recurrence after four years of age was found by Chotigavanichaya et al. in 2002 and by Van Greunen and Firth in 2022, who reported the prevalence of recurrence rising from 46% to 91% and from 25% to 67% before and after this threshold, respectively [5,17]. A recent meta-analysis on 63 studies about ITV and LOTV (1672 knees in 1234 patients) found a threshold of 4.5 years with the recurrence rate rising from 5% to 37% before and after this age [8]. Moreover, by focusing the analysis only on the cases that reached full skeletal maturity, patients that required no further surgical revision were all treated before age four. As a result, we conclude that treatment is most effective below 4 years of age, with a significant rise in recurrence rates between ages 4 and 5, and a high likelihood of recurrence if surgery is performed after age 5.

A notable finding was that the majority of recurrences occurred within 2.5 years post-surgery, aligning with a recent meta-analysis suggesting that recurrence could be missed with shorter follow-up periods [8]. Therefore, it is crucial for future ITV treatment studies to adhere to this minimum follow-up cutoff to correctly estimate recurrence rates.

The association between ITV and obesity is inconsistently reported by authors from different continents, with a prevalence ranging from 30% to 60% [2]. In the past, some authors have hypothesized a biomechanical etiology for ITV, identifying the overload of the medial portion of the physis in obese children as a risk factor [18]. More recently, the association between ITV and obesity was questioned, especially in African, African-American, and African-Caribbean children [19]. The present study, despite finding a high prevalence of obese patients (76%), observed no association between obesity and the prevalence of recurrence. Moreover, unilateral or bilateral involvement was independent from the presence or absence of overweight. In summary, obesity is a very frequent comorbidity in children with ITV treated at an Italian pediatric orthopedic referral center, but its role in pathogenesis is unclear and its presence does not seem to significantly affect surgical outcome.

The present study found a higher prevalence of females among children with ITV (69%), as previously reported by many authors [8,20,21,22]. Of note is the influence of sex on surgical outcome. The higher prevalence of recurrence in females (70%) than in males (25%) seems sufficiently explained by the higher mean age at diagnosis and surgery in girls. To our knowledge, this finding has never been noted by other authors and may be a consequence of the small number of patients included. However, we recommend that primary care pediatricians take special care to avoid a diagnostic delay in girls with ITV.

We found that the single-cut OWHTO with a press-fit cancellous strut bone allograft is effective in achieving a global correction of the axis in one stage, with an average of 15° and a maximum of 25–30°. Unfortunately, beyond the age of 5, we encountered cases with deformities reaching up to 40–45°. Therefore, we can conclude that this technique may still be insufficient to normalize the most severe cases. A possible strategy to reduce the risk and the severity of the recurrence is to plan and perform the osteotomy with initial overcorrection. Eamsobhana et al. found that overcorrection by more than 13° valgus nearly halved the recurrence rate, though insignificantly, due to sample size [23]. Hence, planning interventions with overcorrection may reduce recurrence frequency and severity. Overcorrection can be achieved through gradual correction (with an external fixator) or acute correction through double osteotomies. Our study does not currently allow us to evaluate this hypothesis, although recent studies have reported that acute correction via double osteotomy can be performed without significant risks of vascular stress or compartmental syndrome [24,25,26,27]. On the other side, while gradual correction via the external fixator is a suitable option, its associated higher risk of complications and, notably, the considerable psychological distress it may cause in children warrant consideration [28,29,30,31,32,33]. Another potential contributing factor to our high recurrence rate could be the use of the cancellous strut bone allograft. While it is more accessible in our hospital, easily moldable and adaptable to tibial size, and shows faster and more complete osteointegration, its lower mechanical strength may lead to compression and collapse, resulting in initial correction loss despite proper cast immobilization. Consequently, we have recently transitioned to cortical-cancellous allografts for their superior initial stability. Pre-shaping through virtual surgical planning and graft customization can further enhance initial correction stability and reduce the risk of under-correction [27,34]. Nevertheless, further research is needed to validate this hypothesis. Other strategies may involve the use of autologous bone grafts, particularly the distally removed fibular cylinder, or even bone cement to aid in leg axis correction. However, caution is warranted with options like screw-plates, as while they may enhance immediate mechanical stability, they can also elevate the risk of complications such as compartment syndrome and issues related to wound closure or hardware-related infections.

Among preoperative radiographic variables, Langenskiöld classification showed the highest influence on recurrence rate. However, as already highlighted by many authors, the six traditional stages are improvable: from stage IV upward the recurrence rate is always very high, while stages II and III would require more thorough stratification to estimate the risk of recurrence [35,36,37]. In our series, all patients who did not experience recurrence had a Langenskiöld stage of III or lower. Conversely, patients with a stage beyond grade III invariably faced recurrence due to irreparable damage, including bony bridging and permanent growth arrest of the proximal tibial physis. Therefore, simultaneous lateral hemiepiphysiodesis of the proximal tibia serves as an additional strategy to mitigate recurrence risk, despite the potential for overall tibial shortening [26]. Therefore, it is essential to monitor for potential additional interventions, such as contralateral tibial epiphysiodesis or ipsilateral tibial lengthening close to the end of growth, to mitigate leg length discrepancy, especially in unilateral infantile tibia vara. The role of associated femoral deformity remains to be understood. In our case series, we observed significant compensatory femoral valgus in 9% of preoperative cases, which does not correct spontaneously. We are not aware of the role of a possible alteration of the joint line in this regard.

The response rate to the Patient’s Reported Outcome Measures (PROMs) was low (24%). As other authors observed, the long follow-up and the prolonged time since the last orthopedic evaluation may explain the high prevalence of non-responders [38]. Moreover, the heterogeneity in the number of total surgical procedures and in the age at the time of questionnaire completion prevented an adequate analysis of the functional outcome among responders.

### Limitations

Our study’s strength lies in its analysis of a case series that was from a single center, sufficiently large and uniform in pathology and surgical technique, with a high minimum follow-up period, and analyzed by independent observers. However, the study has limitations. Firstly, its retrospective nature, with data collected over a period of more than 20 years, limits the strength of conclusions that can be drawn. The number of cases, while higher than the average reported in the literature (typically around 20 cases), remains insufficient to develop accurate predictive models incorporating multiple variables.

Since the study was retrospective and all the cases meeting the eligibility criteria were included, no sample size estimation was performed before the beginning of the study. However, the present series effectively confirmed the findings from a recent systematic literature review, which identified a statistically significant difference in the prevalence of recurrence in patients treated before 4.5 years (6%) and after that age (58%) with this technique [8]. Given these characteristics, a sample of 14 cases per group is sufficient to demonstrate a difference in recurrence rate with an alpha error of 5% and a power of 80%.

The absence of a control group treated with different surgical techniques also limits the conclusions that can be drawn from this study. Nonetheless, we believe that insights gained from extensive case analyses in this rare deformity could contribute to proposing future prospective, multicenter, and comparative studies to enhance the prognosis and treatment of this condition.

The assessment of functional outcomes in the patient cohort was insufficient due to the high rate of non-responders (76%). However, for such a rare condition, conducting a longitudinal evaluation of all patients from diagnosis to skeletal maturity is challenging. Referral hospitals need to acquire clinical tools to systematically assess the clinical and functional parameters of patients with rare deformities both before treatment and during follow-up.

## 5. Conclusions

Our study emphasizes the critical role of early diagnosis and treatment for ITV, ideally before age 4 and with Langenskiöld stage III or lower, to achieve stable correction through isolated proximal OWHTO. However, this treatment alone falls short in achieving complete and stable correction in children with late, high-grade, neglected presentations. For such cases, while waiting for further evidence, combined, multiple level, acute or gradual, and/or staged correction should be considered.

## Figures and Tables

**Table 1:** Detailed information on demographic, preoperative, and postoperative variables for the entire cohort and compared between cases with and without recurrence. Quantitative variables with normal distribution are reported as mean ± standard deviation, while those with non-normal distribution are reported as median (first quartile–third quartile). Categorical variables are reported as ratio or percentage.

Variable	Overall	No Recurrence	Recurrence	*p*-Value
Patients (knees)	29 (39)	12 (17)	17 (22)	
Follow-up (years)	7.4 (IQR 4.1–10.6)	8.1 (IQR 3.8–10.0)	7.3 (IQR 4.3–10.6)	0.643
Age at surgery (years)	5.6 ± 1.9	4.2 ± 0.6	6.8 ± 1.7	<0.001
Sex (males/females)	12/27	9/8	3/19	0.008
Side (mono-/bilateral)	16/23	8/9	8/14	0.501
Obesity	74%	71%	77%	0.636
First evaluation-surgery (years)	0.9 ± 0.6	0.9 ± 0.7	0.8 ± 0.4	0.597
Langenskiöld stage	I: 15%	I: 31%	I: 0%	0.008
	II: 25%	II: 50%	II: 11%	
	III: 32%	III: 19%	III: 44%	
	IV: 21%	IV: 0%	IV: 39%	
	V: 3%	V: 0%	V: 6%	
	VI: 0%	VI: 0%	VI: 0%	
MAD (mm)	−53 ± 11	−51 ± 11	−57 ± 11	0.434
aTFA (°)	−16 ± 8	−20 ± 9	−16 ± 7	0.196
TMDA (°)	20 ± 5	17 ± 4	22 ± 5	0.006
Postop. TMDA (°)	6 ± 8	2 ± 6	9 ± 8	0.003
Δ TMDA (°)	−14 ± 6	−15 ± 6	−13 ± 6	0.303
MPDA (°)	46 (IQR 43–50)	45 (IQR 44–50)	47 (IQR 43–50)	0.786
Postop. MPDA (°)	38 (IQR 30–44)	39 (IQR 34–44)	38 (IQR 30–44)	0.520
Δ MPDA (°)	−5 (IQR −17–2)	−6 (IQR −15–2)	−5 (IQR −18–3)	0.718
FC–TS (°)	68 ± 7	71 ± 5	66 ± 7	0.021
Postop. FC–TS (°)	85 ± 7	89 ± 8	81 ± 5	0.002
Δ FC–TS (°)	21 ± 7	19 ± 4	23 ± 9	0.163
mLDFA (°)	93 ± 5	94 ± 3	90 ± 10	0.260
LDTA (°)	93 ± 3	94 ± 3	92 ± 2	0.227
MPTA (°)	71 ± 7	75 ± 3	69 ± 8	0.011
Postop. MPTA (°)	86 ± 6	89 ± 2	83 ± 7	0.004
Δ MPTA (°)	14 ± 5	13 ± 4	14 ± 6	0.543

MAD = mechanical axis distance; aTFA = anatomic tibiofemoral angle; TMDA = proximal tibial metaphyseal–diaphyseal angle (or Drennan–Levine angle); MPDA = angle of depression of the medial tibial plateau; FC–TS = femoral condyle to tibial shaft angle; mLDFA = mechanical lateral distal femoral angle; LDTA = lateral distal tibial angle; MPTA = medial proximal tibial angle; Δ = difference between preoperative and immediate postoperative angles; IQR = interquartile range reporting both values of the first quartile and the third quartile.

## Data Availability

Data are available from the corresponding author upon reasonable request.
